# Supplementary material for: Model composition through model reduction: a combined model of CD95 and NF-κB signaling pathways
Source: BMC Syst Biol. 2013 Feb 15;7:13. doi: 10.1186/1752-0509-7-13 (PMC3626841; doi:10.1186/1752-0509-7-13)
Supplement: Additional file 1: Table 1S — Notations of parameters used in the paper and in the original models. Table 2S. Table of all reactions (excepting degradation) and rate constants in the composite model. Table 3S. Table of degraded species and kinetic laws of degradation in the composite model. Table 4S. Table of nonzero initial concentrations in the composite model. Table 5S. Steady state analysis of the Bentele’s model and the composite model. Species. Table 6S. Steady state analysis of the Neumann’s model and the composite model, Table 7S. Calculation of the mean sensitivity for the investigated apoptosis models. Table 8S. Analysis of predictions regarding apoptosis in HeLa cells as formulated by Neumann et al. Table 9S. Predictions of the models for SKW 6.4 cells. Table 10S. Parametric constraints of the models by Bentele et al. and Neumann et al. [file 1752-0509-7-13-S1.pdf]

# Supplementary tables

## Table 1S – Notations of parameters used in the paper and in the original models

| Parameters notation in the paper | Parameters notation by Bentele *et al.*, 2004 |
|---|---|
| $f_{degr}$ | $f_{degrad}$ |
| $k_{Apop}$ | $k\_Apoptosome$ |
| $k_d$ | $K\_DEGRAD$ |
| $k_{dd}$ | $K\_DEGRAD\_deathSub$ |
| $k_{ds}$ | $K\_DEGRAD\_steady$ |
| $k_{DISC\_FLIP}$ | $k\_DISC\_FLIP$ |
| $k_{DISC\_pro8}$ | $k\_DISC\_procas8$ |
| $k_{DFp8}$ | $k\_DISC\_FLIP\_to\_cas8\_IM$ |
| $k_{LR}$ | $k\_LR$ |
| $k_{32}$ | $k\_cas\_3\_2$ |
| $k_{36}$ | $k\_cas\_3\_6$ |
| $k_{38}$ | $0.145 \cdot k\_cas\_6\_8$ |
| $k_{68}$ | $k\_cas\_6\_8$ |
| $k_{78}$ | $k\_cas\_8\_7$ |
| $k_{83}$ | $k\_cas\_8\_3$ |
| $k_{3act}$ | $0.18 \cdot k\_cas7\_apop\_activity$ |
| $k_{36act}$ | $k\_cas36\_apop\_activity$ |
| $k_{39IAP}$ | $k\_cas39\_IAP$ |
| $k_{7act}$ | $k\_cas7\_apop\_activity$ |
| $k_{8Bid}$ | $k\_cas8\_Bid$ |
| $Km_{32}$ | $Km\_cas\_3\_2$ |
| $Km_{36}$ | $Km\_cas\_3\_69$ |
| $Km_{367act}$ | $Km\_cas367\_apop\_activity$ |
| $Km_{38}$ | $Km\_cas\_6\_8$ |
| $Km_{68}$ | $Km\_cas\_6\_8$ |
| $Km_{78}$ | $Km\_cas\_8\_7$ |
| $Km_{8Bid}$ | $Km\_cas28\_Bid$ |
| $Km_{893}$ | $Km\_cas\_89\_3$ |
| $x_{aa}$ | $x_{apop\ activity}$ |
| | |
| **Parameters notation in the paper** | **Parameters notation by Neumann *et al.*, 2004** |
| $k_{p43-FLIP\_IKK}$ | $k13$ |

**Table 2S – Table of all reactions (excepting degradation) and rate constants in the composite model**

| № | Reactions | Kinetic laws | Rate constants ($nM^{-1}min^{-1}$; $min^{-1}$) | Comments[1] |
|---|---|---|---|---|
| nr1 | CD95L + CD95R:FADD → DISC | $n_{k1} \cdot C_{CD95L} \cdot C_{CD95R}$ | 1.0 | $k1$, Neumann *et al.*, 2010 |
| nr2 | pro8 + DISC → DISC:pro8 | $n_{k2} \cdot C_{DISC} \cdot C_{pro8}$ | $1.277248E-4$ | $k2$, Neumann *et al.*, 2010 |
| nr3 | DISC + FLIPL → DISC:FLIPL | $n_{k3} \cdot C_{DISC} \cdot C_{FLIPL}$ | 0.6693316 | $k3$, Neumann *et al.*, 2010 |
| nr5 | DISC:pro8 + pro8 → 2 x p43p41 | $n_{k5} \cdot C_{DISC:pro8} \cdot C_{pro8}$ | $5.946569E-4$ | $k5$, Neumann *et al.*, 2010 |
| nr6 | DISC:pro8 + FLIPL → p43-FLIP | $n_{k6} \cdot C_{DISC:pro8} \cdot C_{FLIPL}$ | 0.9999999 | $k6$, Neumann *et al.*, 2010 |
| nr7 | DISC:pro8 + FLIPS → DISC:pro8:FLIPS | $n_{k7} \cdot C_{DISC:pro8} \cdot C_{FLIPS}$ | 0.8875063 | $k7$, Neumann *et al.*, 2010 |
| nr8 | DISC:FLIPL + pro8 → p43FLIP | $n_{k5} \cdot C_{DISC:FLIPL} \cdot C_{pro8}$ | $5.946569E-4$ | $k5$, Neumann *et al.*, 2010 |
| nr9 | DISC:FLIPL + FLIPL → DISC:$FLIPL_2$ | $n_{k6} \cdot C_{DISC:FLIPL} \cdot C_{FLIPL}$ | 0.9999999 | $k6$, Neumann *et al.*, 2010 |
| nr10 | DISC:FLIPL + FLIPS → DISC:FLIPL:FLIPS | $n_{k7} \cdot C_{DISC:FLIPL} \cdot C_{FLIPS}$ | 0.8875063 | $k7$, Neumann *et al.*, 2010 |
| nr14 | 2 x p43p41 → casp8 | $n_{k8} \cdot C^2_{p43p41}$ | $8.044378E-4$ | $k8$, Neumann *et al.*, 2010 |
| nr15_m | pro3 –casp9→ casp3 | $n_{k9} \cdot C_{casp9} \cdot C_{pro3}$ | 0.04920673 | **Fitted value**,<br>$k9 = 0.002249759$, Neumann *et al.*, 2010 |
| nr16 | pro8 -casp3→ p43p41 | $n_{k10} \cdot C_{pro8} \cdot C_{casp3}$ | 0.01205258 | **Fitted value**,<br>$k10 = 0.1205258$, Neumann *et al.*, 2010 |
| nr19 | p43FLIP → p43-FLIP:IKK* | $n_{k13} \cdot IKK_0 \cdot C_{p43FLIP}$ | $n_{k13} = 7.20426E-4$,<br>$IKK_0 = 5.772825$ | $k13$, $C_{IKK}(0)$, Neumann *et al.*, 2010 |
| nr20 | NF-κB:IκB -p43-FLIP:IKK→ NF-κB:IκB-P | $n_{k14} \cdot C_{\mathrm{NF\kappa B:I\kappa B}} \cdot C_{p43FLIP:IKK^*}$ | 0.3588224 | $k14$, Neumann *et al.*, 2010 |
| nr21 | NF-κB:IκB-P → NF-κB* | $n_{k15} \cdot C_{\mathrm{NF\kappa B:I\kappa BP}}$ | 3.684162 | $k15$, Neumann *et al.*, 2010 |
| br6*_m | pro9 -tBid→ casp9 | $b_{k6} \cdot C_{tBid} \cdot C_{pro9}$ | 0.06310456 | – |
| br7* | Bid -casp8→ tBid | $b_{k7} \cdot C_{casp8} \cdot C_{Bid}$ | $6.0004E-4$ | **Fitted value**,<br>$k_{8Bid}/Km_{8Bid} = 5.3325E-4$,<br>the reduced Bentele's model |
| br8* | pro2 -casp3→ (cleavage) | $\frac{b_{k8} \cdot C_{casp3} \cdot C_{pro2}}{b_{Km8} + C_{pro2}}$ | $b_{k8} = 15.63123994$,<br>$b_{Km8} = 55.57400642$ | **Fitted values**,<br>$k_{32} = 0.18137$, $Km_{32} = 55.574$,<br>Bentele *et al.*, 2004 |
| br9* | pro3 -casp8→ casp3 | $\frac{b_{k9} \cdot C_{casp8} \cdot C_{pro3}}{b_{Km9} + C_{pro3}}$ | $b_{k9} = 0.0$ (HeLa) or<br>0.27246 (SKW 6.4),<br>$b_{Km9} = 1.03542544$ | **Fitted values**,<br>$k_{83} = 1.90358$, $Km_{893} = 89.02911547$,<br>Bentele *et al.*, 2004 |
| br10* | pro7 -casp8→ (cleavage) | $b_{k10} \cdot C_{casp8} \cdot C_{pro7}$ | 10.89517864 | **Fitted value**,<br>$k_{78}/Km_{78} = 0.01016$, the reduced Bentele's model |
| br11* | PARP –casp3→ cPARP | $b_{k11} \cdot C_{casp7} \cdot C_{PARP}$ | 73.49560485 | **Fitted value**,<br>$0.18 \cdot k_{7act}/Km_{367act} = 0.00824$,<br>the reduced Bentele's model |
| br12* | casp3 + IAP → (inhibition) | $b_{k12} \cdot C_{casp3} \cdot C_{IAP}$ | 0.01 | **Fitted value**,<br>$k_{39IAP} = 45824.409 \cdot 5 \cdot 10^{-5}$, Bentele *et al.*, 2004 |
| br13* | -casp3→ Apoptotic activity | $b_{k13} \cdot (1.0 - x_{aa}) \cdot C_{casp3}$ | 5956.81008868 | **Fitted value**,<br>$(k_{36act} + 0.18 \cdot k_{7act})/Km_{367act} = 0.01053$,<br>Bentele et al., 2004 |

[1] The column contains basic parameters or expressions (and their values if they were refitted) in the combined models.

## Table 3S – Table of degraded species and kinetic laws of degradation in the composite model

| Degraded species | Degradation rates | Rate constants (min$^{-1}$) |
|---|---|---|
| pro2, pro3, pro8, DISC:pro8, DISC:pro8:FLIPS, pro9, Bid, PARP | $f_{degr} = degr \cdot x_{aa}^2$ | $degr = \begin{cases} 0.0, & \text{HeLa cells}, \\ 0.0542, & \text{SKW 6.4 cells}, \quad C_{CD95L} = 5\,\mu g/ml, \\ 0.0084, & \text{SKW 6.4 cells}, \quad C_{CD95L} = 200\,ng/ml, \\ 0.0028, & \text{SKW 6.4 cells}, \quad 1\ ng/ml \le C_{CD95L} \le 100\,ng/ml. \end{cases}$ |
| casp3 | $f_{degr} = degr \cdot x_{aa}^2 + n_{k12}$ | $n_{k12} = 0.1502914$, ($k12$, Neumann *et al.*, 2010) |
| casp8 | $f_{degr} = degr \cdot x_{aa}^2 + n_{k11}$ | $n_{k11} = 0.02891451$, ($k11$, Neumann *et al.*, 2010) |
| casp9 | $f_{degr} = degr \cdot x_{aa}^2 + degr_{casp9}$ | $degr_{casp9} = 0.10025628$ |
| cPARP | $f_{degr} = degr_{cPARP}$ | 0.01748, (K_DEGRAD_PARP, Bentele *et al.*, 2004) |
| tBid | $f_{degr} = degr_{tBid}$ | 0.04999612 |
| p43-FLIP:IKK* | $f_{degr} = n_{k16}$ | 0.02229912, ($k16$, Neumann *et al.*, 2010) |
| Apoptotic activity ($x_{aa}$) | $degr_{apop\ activity}$ | 0.00219407 |

## Table 4S – Table of nonzero initial concentrations in the composite model

| Species | Initial concentrations (nM) | | | |
|---|---|---|---|---|
| | **Neumann *et al.*, 2010 HeLa cells** | **Bentele *et al.*, 2004 SKW 6.4 cells** | **Composite model HeLa cells** | **Composite model SKW 6.4 cells** |
| CD95L | 113.22, 37.74, 18.87 | 1990.0, 79.6 | 113.22, 37.74, 18.87 | 1990.0, 79.6 |
| CD95R | – | 442.820768294033 | – | – |
| CD95R:FADD | 91.26592 | 0.0 | 91.26592 | 611.6891578799691 |
| procaspase-2 | – | 157.644193512676 | 157.644193512676 | 157.644193512676 |
| procaspase-3 | 1.443404 | 112.45433410827 | 14.43404 | 2.3438636537313413 |
| procaspase-7 | – | 18.7933134063988 | 18.7933134063988 | 18.7933134063988 |
| procaspase-8 | 64.47652 | 442.820768294033 | 64.47652 | 350.0248656584318 |
| procaspase-9 | – | 245.101295250747 | 2.9090736162783806 | 245.101295250747 |
| FLIPL | 7.398562 | 65.0213661020702 | 7.398562 | 7.398562 |
| FLIPS | 5.083923 | 65.0213661020702 | 5.083923 | 70.44906883596883 |
| IAP | – | 12.2160965349275 | 1.221610 | 1.221610 |
| Bid | – | 231.760433964353 | 5.003142624870996 | 231.760433964353 |
| IKK | 5.772825 | – | 5.772825 | 5.772825 |
| NF-κB:IκB | 4.739546 | – | 4.739546 | 4.739546 |
| PARP | – | 11.1615188752353 | 11.1615188752353 | 11.1615188752353 |

### Table 5S – Steady state analysis of the Bentele's model and the composite model

| Species | Steady state values, anti-CD95 = 200 ng/ml | | | Steady state values, anti-CD95 = 5 μg/ml | | |
|---|---|---|---|---|---|---|
| | Original model | Reduced model | Composite model | Original model | Reduced model | Composite model |
| procaspase-2 | 0.0000374 | 0.0000316 | 2.4468579 | 0.0013440 | 0.0013602 | 0.0000096 |
| procaspase-3 | – | – | – | 0.0 | 0.0 | 0.0 |
| procaspase-7 | – | – | – | 0.0 | 0.0 | 0.0 |
| procaspase-8 | 25.1700455 | 27.2240392 | 14.9514288 (total value) | 0.0000016 | 0.0 | 0.0000367 (total value) |
| procaspase-9 | 0.0 | 0.0 | 0.0 | – | – | – |
| caspase-8 | 0.0 | 0.0 | 0.0 | 0.0 | 0.0 | 0.0 |
| p43/p41 | 0.0 | 0.0 | 0.0001051 | – | – | – |
| Bid | – | – | – | 0.0411840 | 0.0342991 | 0.0000298 |
| tBid | – | – | – | 0.0901602 | 0.1040935 | 0.0 |
| PARP | 0.0 | 0.0 | 0.0 | 0.0 | 0.0 | 0.0 |
| cPARP | – | – | – | 0.0 | 0.0 | 0.0 |

### Table 6S – Steady state analysis of the Neumann's model and the composite model

| Species | Steady state values, anti-CD95 = 1500 ng/ml | | | Steady state values, anti-CD95 = 500 ng/ml | | | Steady state values, anti-CD95 = 250 ng/ml | | |
|---|---|---|---|---|---|---|---|---|---|
| | Original model | Reduced model | Composite model | Original model | Reduced model | Composite model | Original model | Reduced model | Composite model |
| procaspase-8 (total) | 12.0752771 | 12.0751903 | 12.0181928 | 2.6562426 | 2.6562649 | 2.3208092 | 9.6046242 | 9.6052138 | 13.2926881 |
| p43/p41 | 0.0010204 | 0.0010204 | 0.0000891 | 0.0009555 | 0.0009555 | 0.0000948 | 0.0008922 | 0.0008922 | 0.0000883 |
| caspase-8 | 0.0 | 0.0 | 0.0 | 0.0 | 0.0 | 0.0 | 0.0 | 0.0 | 0.0 |
| procaspase-3 | 0.1992635 | 0.1992525 | 3.8521010 | 0.1350020 | 0.1349977 | 3.7249153 | 0.1721551 | 0.1721569 | 3.8516549 |
| caspase-3 | 0.0 | 0.0 | 0.0 | 0.0 | 0.0 | 0.0 | 0.0 | 0.0 | 0.0 |
| IκB | 0.0 | 0.0 | 0.0 | 0.0000020 | 0.0000020 | 0.0000020 | 0.1519007 | 0.1526973 | 0.1526973 |
| IκB-P | 0.0 | 0.0 | 0.0 | 0.0 | 0.0 | 0.0 | 0.0 | 0.0 | 0.0 |
| p43-FLIP | 0.0 | 0.0 | 0.0 | 0.0 | 0.0 | 0.0 | 0.0 | 0.0 | 0.0 |

### Table 7S – Calculation of the mean sensitivity for the investigated apoptosis models

| Cell lines, anti-CD95 concentrations | The model by Bentele *et al.* | | | The model by Neumann *et al.* | | | The composite model, all parameters |
|---|---|---|---|---|---|---|---|
| | Original model | | Reduced model | Original model | | Reduced model | |
| | All parameters | Retained parameters | | All parameters | Retained parameters | | |
| SKW 6.4, 5 μg/ml | −34.49 | −33.79 | −29.72 | – | – | – | −146.84 |
| SKW 6.4, 200 ng/ml | −25.64 | −24.95 | −38.97 | – | – | – | −114.13 |
| HeLa, 1500 ng/ml | – | – | – | −30.71 | −30.42 | −30.29 | −30.21 |
| HeLa, 500 ng/ml | – | – | – | −79.85 | −79.52 | −78.94 | −79.88 |
| HeLa, 250 ng/ml | – | – | – | −88.05 | −87.60 | −88.70 | −97.69 |

**Table 8S – Analysis of predictions regarding apoptosis in HeLa cells as formulated by Neumann *et al.***

| №[2] | Behavior of the model by Neumann *et al.* | Behavior of the reduced model | The composite model behavior | Predictions by Neumann *et al.*[3] |
|---|---|---|---|---|
| 1* | 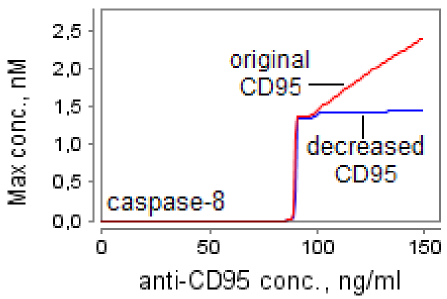  | 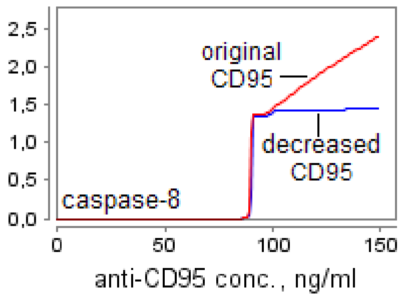  | 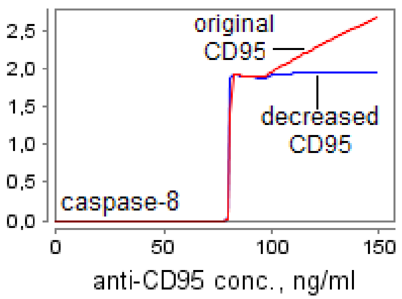  | The concentration of anti-CD95 required for the apoptosis induction (the apoptotic threshold), is within the range of 30-100 ng/ml. This range remains the same for CD95 decreased by about 12-fold.<br><br>The simulation time, which we used to reproduce this prediction, was 60 hours. |
| 2* | 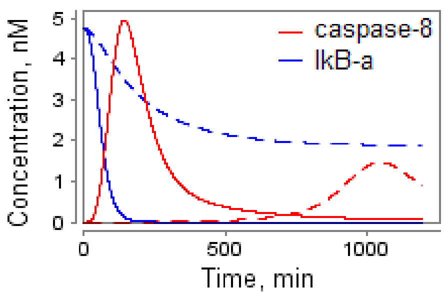  | 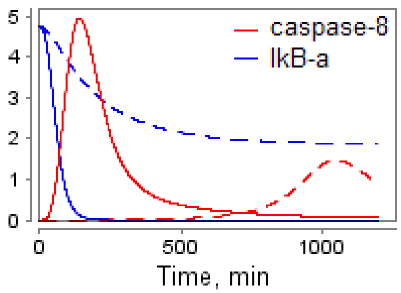  | 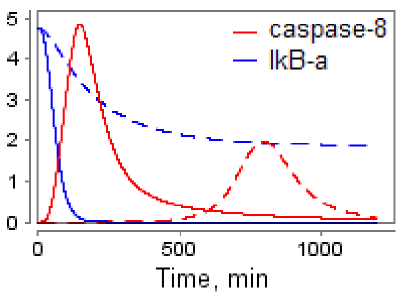  | The decreased receptor number results in impairment of both CD95- and NF-κB-signaling pathways. To test this prediction, Neumann *et al.* considered levels of caspases-8 cleavage and IκB-α degradation for the original (solid lines) amount of CD95 and the amount decreased by about 12-fold (dashed lines). The concentration of anti-CD95 was 500 ng/ml. |
| 3* | 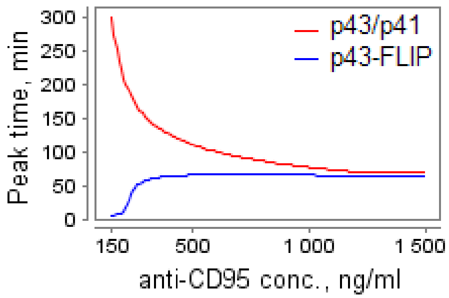  | 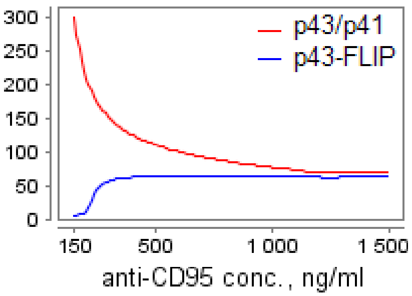  | 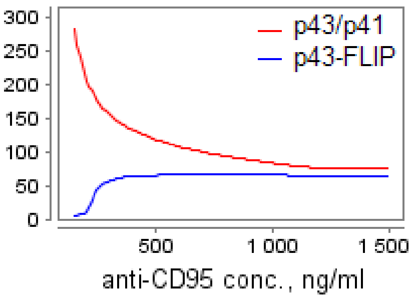  | Along with increasing the concentration of anti-CD95 from 500 ng/ml to 1500 ng/ml, p43/p41 peaks earlier, while there is almost no difference for p43-FLIP. |
| 4 | 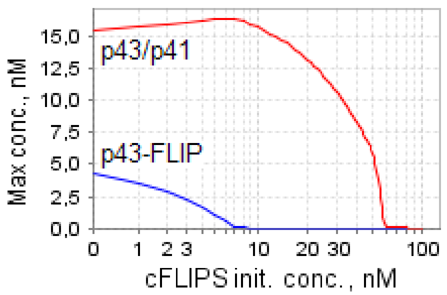  | 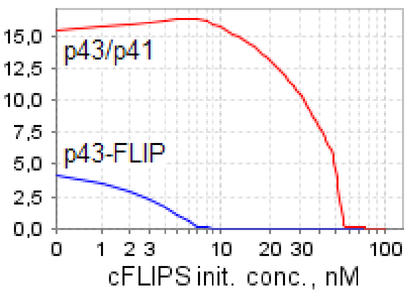  | 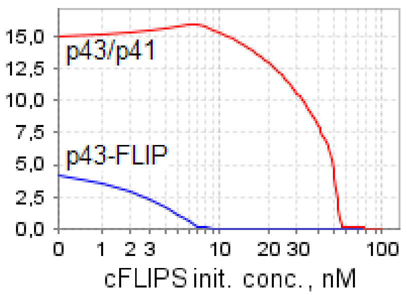  | Increased concentrations of cFLIPS inhibit both apoptotic and NF-κB pathways, although p43-FLIP generation is inhibited at a lower threshold than p43/p41 generation. |

[2] All the predictions marked with an asterisk were experimentally tested by Neumann *et al.* and confirmed, unless otherwise noted.
[3] The The simulation time in predictions 3-7 was 360 min. The concentration of anti-CD95 considered by the authors in predictions 4-7 was 1000 ng/ml.

**Table 8S (continuation)**

| № | Behavior of the model by Neumann *et al.* | Behavior of the reduced model | The composite model behavior | Predictions by Neumann *et al.* |
|---|---|---|---|---|
| 5* | 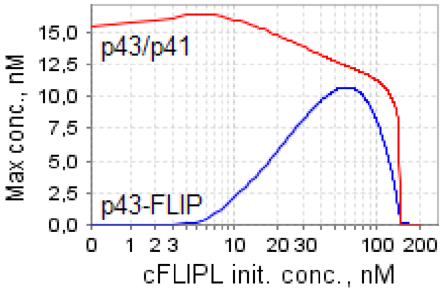 | 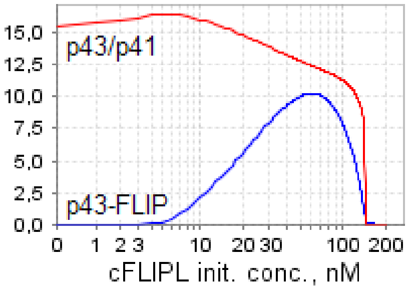 | 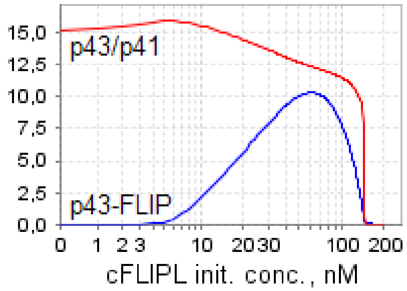 | Increasing the concentration of cFLIPL leads to a steep increase in p43-FLIP generation until it reaches a maximum, after which the curve drops. Lowered levels of cFLIPL result in very little p43-FLIP but almost unchanged levels of p43/p41.<br><br>At very high concentrations of cFLIPL no p43-FLIP is generated. This drop-off was not observed experimentally by the authors. |
| 6* | 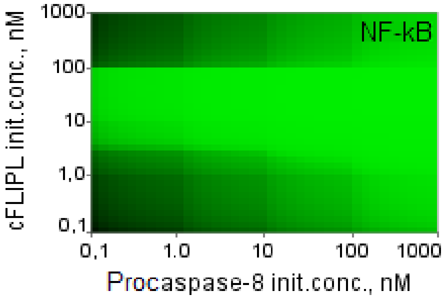 | 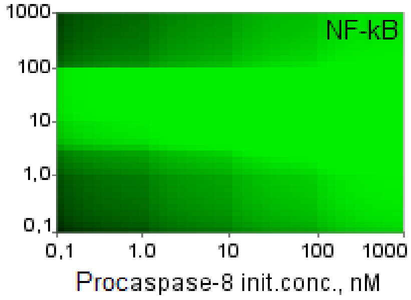 | 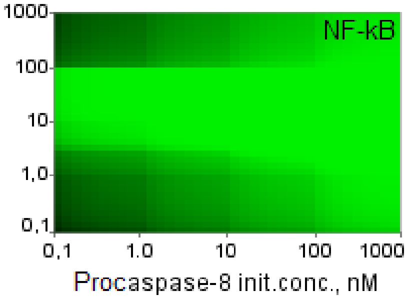 | Only an intermediate level of cFLIPL promotes NF-kB activation. Decreased levels of procaspase-8 lead to a significantly lower amount of p43-FLIP and, subsequently, NF-κB. The figures show of logarithmic dependence of the maximal NF-κB concentration on the initial values of procaspase-8 and cFLIPL |
| 7* | 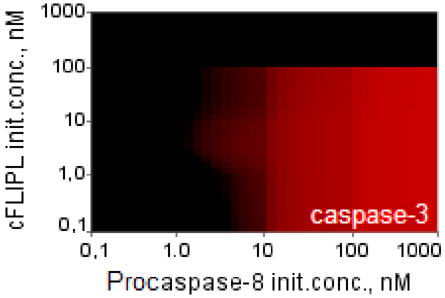 | 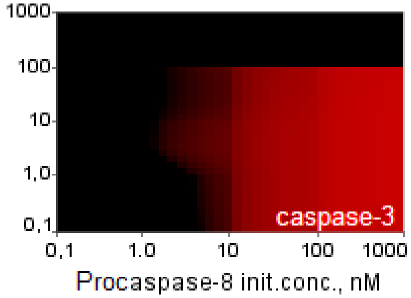 | 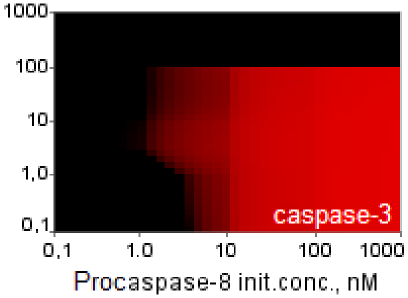 | High cFLIPL or low procaspase-8 concentrations cause suppression of apoptosis. The figures show the same dependence as considered in the previous prediction, but with caspases-3 instead of NF-κB. |

### Table 9S – Predictions of the models for SKW 6.4 cells

| № | Behavior of the model by Bentele *et al.* | Behavior of the reduced model | The composite model behavior | Experimental observations by Bentele *et al.* and predictions of the models[4] |
|---|---|---|---|---|
| 1 | 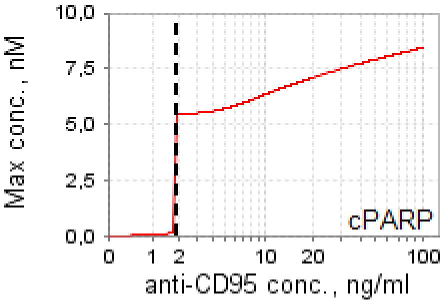 | 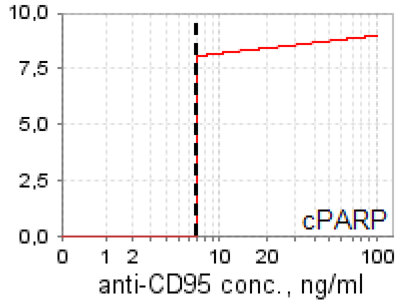 | 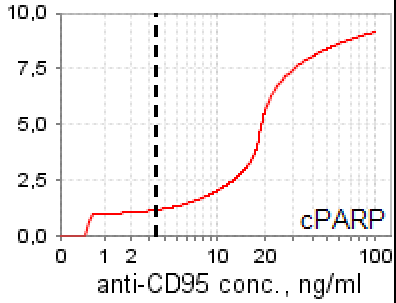 | **Experiments by Bentele *et al.*:**<br>– for 1 ng/ml of anti-CD95, PARP cleavage was not observed;<br>– the measured death rate for 10 ng/ml of anti-CD95 was 20-30%.<br>**Original and reduced models:**<br>– the apoptotic threshold[5] is 1.9 and 6.9 ng/ml for the original and reduced models, respectively;<br>– cPARP concentration rises dramatically within an extremely narrow interval of anti-CD95 levels overcoming the apoptotic threshold.<br>**Composite model:**<br>– the apoptotic threshold is 3.5 ng/ml;<br>– cPARP concentration rises in a smooth manner along with the increase of anti-CD95 level. |
| 2 | 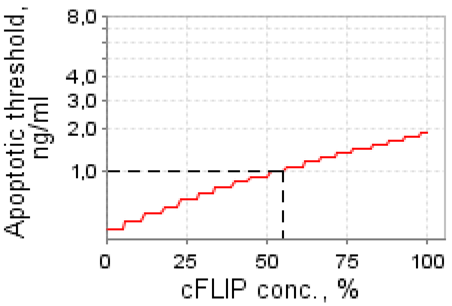 | 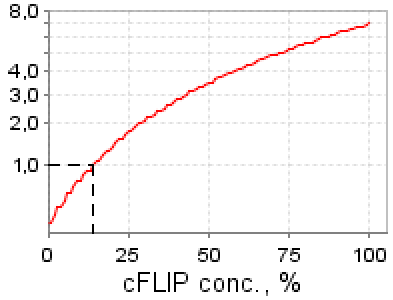 | 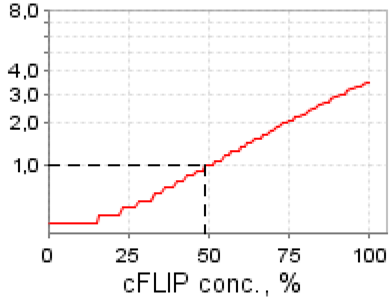 | **Experiments by Bentele *et al.*:** down-regulation of cFLIP in SKW 6.4 cells by addition of cyclohexamide resulted in cell death (40% for 1 day) already upon 1 ng/ml of anti-CD95. The level of cFLIP was decreased to 70%.<br>**All models:**<br>– the apoptotic threshold is highly sensitive to the concentration of cFLIP;<br>– decreasing the initial concentration of cFLIP by more than 51%, 86% and 49% for the original, reduced and composite models, respectively, leads to cell death upon stimulation by 1 ng/ml of anti-CD95. |
| 3 | 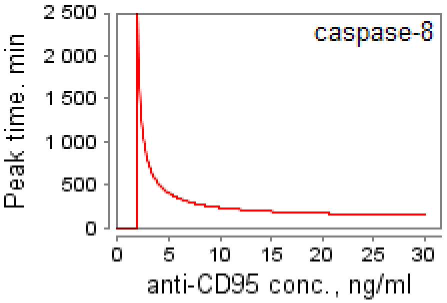 | 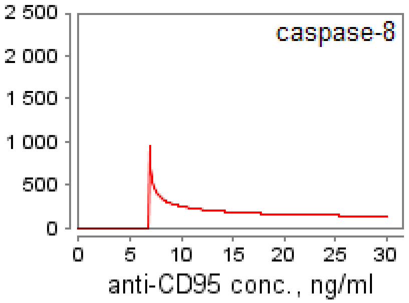 | 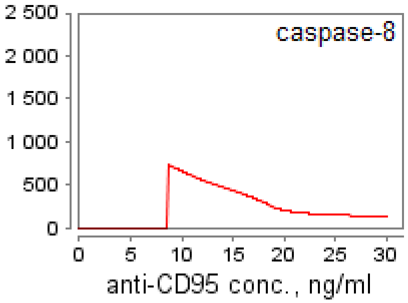 | **Experiments by Bentele *et al.*:** in the 10 ng/ml activation scenario, a significant increase of caspase-8 was observed after more than 4 hours.<br>**All models:** anti-CD95 concentrations which are slightly above the apoptic threshold result in caspase-8 activation after a delay of many hours.<br><br>The figures show peak times of caspase-8 concentration exceeding 0.1% of initial procaspase-8 level. |

[4] The simulation time was 2880 min (2 days) in all predictions.
[5] The apoptotic threshold is the concentration of anti-CD95 after which cPARP amount exceeds 10% of the initial PARP level.

**Table 9S (continuation)**

| № | Behavior of the model by Bentele *et al.* | Behavior of the reduced model | The composite model behavior | Experimental observations by Bentele *et al.* and predictions of the models |
|---|---|---|---|---|
| 4 | 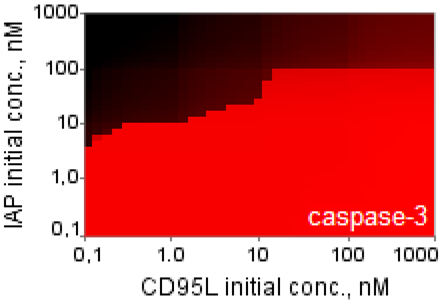 | 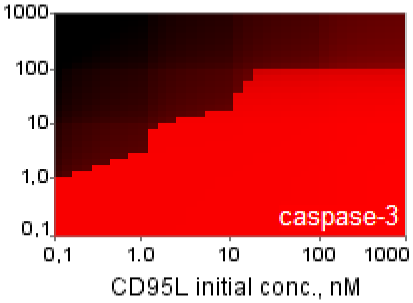 | 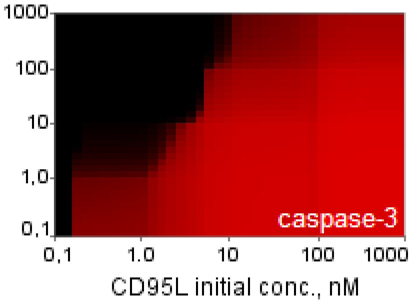 | **Original and reduced models:**<br>– low concentrations of IAP (less than 1 nM) result in complete cell death;<br>– high concentrations of IAP prevent significant increase of caspase-3 even for high concentrations of the ligand.<br>**Composite model:**<br>– low concentrations of IAP (less than 1 nM) block apoptosis for CD95L less than 0.3 nM;<br>– high concentrations of CD95L lead to cell death.<br><br>The figures show logarithmic dependence of the maximal caspases-3 concentration on initial values of IAP and CD95L. |

**Table 10S – Parametric constraints of the models by Bentele *et al.* and Neumann *et al.***

| № | Parametric constraints of the Bentele's model[6] | Corresponding reduction steps |
|---|---|---|
| 1 | $C_{casp7} \approx a \cdot C_{casp3},\ a = const$,<br>$k_{7act} \cdot C_{casp7} \gg k_{36act} \cdot C_{casp3} > k_{36act} \cdot C_{casp6}$,<br>$Km_{367act} \gg (1 - x_{aa})$ | Simplification of the equation of $x_{aa}$ production |
| 2 | $v_{br3} \approx v_{br4} \approx v_{br5} \approx v_{br6} \approx v_{br12} \approx v_{br13}$ | Elimination of quasi-stationary intermediates CD95R:CD95L, DISC:pro8, DISC:pro8$_2$ and DISC:p43/p41. Removal of slow reactions br12 and br13 |
| 3 | $C_{casp6} \approx b \cdot C_{casp3}$, $b = const$ | Deletion of caspase-6 and procaspase-6 |
| 4 | $C_{pro8} \gg Km_{68}$ or $v_{br6} \gg v_{br8}$ | Formation of the linear kinetic law of caspase-8 activation triggered by caspase-3 |
| 5 | $v_{br9} \approx v_{br10}$ | Elimination of the quasi-stationary species DISC:cFLIPL |
| 6 | $C_{FLIPL}(0) = C_{FLIPS}(0)$,<br>reactions br9 and br11 have the same rate constant $k_{DISC\_FLIP}$. | Lumping of cFLIPL and cFLIPS |
| 7 | $v_{br19}$, $v_{casp9\ degr}$ >> $v_{br20}$, $v_{br35}$, $v_{br38}$ | Deletion of slow reactions of apoptosome complex dissociation (br20), caspase-9 inhibition (br35) and casp9:IAP dissociation (br38) |
| 8 | $v_{br15}/(v_{br16} - vbr17\ ) \approx const$ | Elimination of cytochrome *C* |
| 9 | $v_{br18} \approx v_{br19}$ | Elimination of the quasi-stationary apoptosome complex |
| 10 | $v_{br22} > v_{br23}$ | Deletion of reaction br23 (Bid truncation) and caspase-2 |
| 11 | $Km_{8Bid} \gg C_{Bid}$ | Replacement of the Michaelis-Menten kinetics in br22 with the mass action kinetics |
| 12 | $v_{br27} > v_{br28}$,<br>$v_{br29} \gg v_{br30}$ | Removal of the reactions of caspase-3 (br28) and caspase-7 (br30) cleavage triggered by caspase-9 |
| 13 | $v_{br27}, v_{br33}, f_{degr}(x_{aa}) \cdot C_{casp3} \gg v_{br36}$,<br>$v_{br29}, v_{br34}, f_{degr}(x_{aa}) \cdot C_{casp7} \gg v_{br37}$ | Deletion of the slow reactions of casp3:IAP (br36) and casp7:IAP (br37) dissociation |
| 14 | $v_{br33}, v_{br34} \gg v_{br25}, v_{br26}$ | Taking into account the steps described above, we can get only four reactions involving IAP (br33, br34, br25 and br26). At this stage, we can remove reactions br25 and br26, as well as their members Smac and IAP:Smac. |
| 15 | $v_{br32} > v_{br31}$ | Removal of the reaction of PARP cleavage by caspase-3 |
| 16 | $Km_{78} \gg C_{pro7}$ | Replacement of the Michaelis-Menten kinetics in br29 by the mass action kinetics |
| 17 | $Km_{367act} \gg C_{PARP}$,<br>$C_{casp7} \approx a \cdot C_{casp3}$, $a = const$ | Modification of kinetics of br32 |
| | | |
| **№** | **Parametric constraints of the Neumann's model** | **Corresponding reduction steps** |
| 1 | $k2 \cdot C_{pro8}, k3 \cdot C_{FLIPL} \gg k4 \cdot C_{FLIPS}$ | The slow reaction of the DISC binding by FLIPS (nr4) leads to production of a very small amount of DISC:FLIPS. Therefore, all reactions involving this complex (nr4, nr11-nr13) can be removed without any significant effect on the model dynamics. |
| 2 | $C_{IKK} \gg C_{p43FLIP}$ | Setting the constant value of the IKK concentration |

[6]We did not analyze the constraints on degradation rates of species.
